# Site Directed Mutagenesis of Amino Acid Residues at the Active Site of Mouse Aldehyde Oxidase AOX1

**DOI:** 10.1371/journal.pone.0005348

**Published:** 2009-04-29

**Authors:** Silvia Schumann, Mineko Terao, Enrico Garattini, Miguel Saggu, Friedhelm Lendzian, Peter Hildebrandt, Silke Leimkühler

**Affiliations:** 1 Universität Potsdam, Institut für Biochemie and Biologie, Potsdam, Germany; 2 Department of Biochemistry and Molecular Pharmacology, Istituto de Ricerche Farmacologiche, “Mario Negri”, Milano, Italy; 3 Technische Universität Berlin, Institut für Chemie, Berlin, Germany; Cairo University, Egypt

## Abstract

Mouse aldehyde oxidase (mAOX1) forms a homodimer and belongs to the xanthine oxidase family of molybdoenzymes which are characterized by an essential equatorial sulfur ligand coordinated to the molybdenum atom. In general, mammalian AOs are characterized by broad substrate specificity and an yet obscure physiological function. To define the physiological substrates and the enzymatic characteristics of mAOX1, we established a system for the heterologous expression of the enzyme in *Eschericia coli*. The recombinant protein showed spectral features and a range of substrate specificity similar to the native protein purified from mouse liver. The EPR data of recombinant mAOX1 were similar to those of AO from rabbit liver, but differed from the homologous xanthine oxidoreductase enzymes. Site-directed mutagenesis of amino acids Val806, Met884 and Glu1265 at the active site resulted in a drastic decrease in the oxidation of aldehydes with no increase in the oxidation of purine substrates. The double mutant V806E/M884R and the single mutant E1265Q were catalytically inactive enzymes regardless of the aldehyde or purine substrates tested. Our results show that only Glu1265 is essential for the catalytic activity by initiating the base-catalyzed mechanism of substrate oxidation. In addition, it is concluded that the substrate specificity of molybdo-flavoenzymes is more complex and not only defined by the three characterized amino acids in the active site.

## Introduction

The molybdenum cofactor (Moco) is found in a class of widely distributed proteins collectively known as molybdoenzymes. Moco containing enzymes are divided into three separate groups on the basis of structure, cofactor and spectroscopic characteristics: the dimethylsulfoxide (DMSO) reductase, the xanthine oxidase, and the sulfite oxidase family [1]. Members of the xanthine oxidase family comprise xanthine dehydrogenase (XDH, EC 1.17.1. 4), xanthine oxidase (XO, EC 1.17.3.2), and aldehyde oxidase (AO, EC 1.2.3.1). All these proteins are characterized by an equatorial sulfur ligand at the Moco essential for the enzymatic activity [2], and belong to the class of complex molybdo-flavoenzymes (MFEs) containing two nonidentical [2Fe-2S] clusters and FAD as additional cofactors [3], [4].

Vertebrate xanthine oxidoreductases (XOR) are the products of single orthologous genes and are the key enzymes in the catabolism of purines, oxidizing hypoxanthine to xanthine and xanthine to the terminal catabolite uric acid, with the concomitant reduction of NAD^+^ (XDH) or O_2_ (XO) [5].

The gene family of vertebrate AOs is much more complex and the corresponding protein products have been the object of fewer studies. While the human genome is characterized by a single functionally active AO gene (AOX1), the complement of homologous genes in rodents and other mammals is more complex [4]. Marsupials and rodents contain the highest number of functionally active AO genes, which generally cluster on the same chromosomal region at a short distance from one another [4]. The rodent AO gene cluster consists of the human AOX1 orthologue, and three highly related genes named AO homologue-1 (AOH1), -2 (AOH2), and -3 (AOH3) [4], [6]–[8]. In other vertebrates and mammals the number of functionally active loci varies, as a consequence of gene duplication and suppression events [4]. The multiple AO isoforms are expressed tissue-specifically [4], [7], and may recognize distinct substrates and carry out different physiological tasks. The overall level of amino acid identity between AO and XOR proteins is approximately 50%, which indicates that the two proteins originated from a common ancestral precursor [3].

While the biochemical function of XOR is well established, the biochemical and physiological functions of AO are still largely obscure [4]. Monogenic deficits of mammalian AO isoforms have not been reported to date. In humans, AOX1 and XOR do not seem to play a vital role, as genetic deficiencies in the Moco sulfurase (MCSF) gene, causing a defect of both enzymes, are associated with mild symptoms such as the formation of kidney stones [9], [10]. AOs in general are characterized by a broad substrate specificity and play an important role in the metabolism of drugs and xenobiotica [3]. In animals, AOs have a significant toxicological role, detoxifying xenobiotics of wide structural diversity. The enzymes oxidize aromatic aza-heterocycles containing a –CH = N− chemical function (e.g. phtalazine and purines), aromatic or non-aromatic charged aza-heterocycles with a –CH = N^+^ =  moiety (e.g. N^1^-methylnicotinamide and N-methylphthalazinium) or aldehydes, such as benzaldehyde, retinal and vanillin [3]. AO and XOR share some common substrates and the relative selectivity of the two types of enzymes has been systematically reviewed [11]. AO may have a role in the degradation of vitamins like nicotinamide and pyridoxal or in the oxidation of *all-trans* retinaldehyde to *all-trans* retinoic acid, the active metabolite of vitamin A [3]. Upon oxidation of aldehyde substrates, AO produces significant amounts of highly toxic reactive oxygen species, O_2_^.−^ and hydrogen peroxide [12], [13].

The reaction mechanism of substrate oxidation of MFEs has been only described in detail for *Rhodobacter capsulatus* XDH [14], a bacterial XDH sharing high similarities to eukaryotic XOR both at the structural level and on the basis of amino acid sequence identity [15], [16]. In the oxidized enzyme, the metal is in the Mo(VI) oxidation state, bearing an oxo ( = O), an hydroxo (-OH) and an equatorial sulfido ( = S) ligand. Site-directed mutagenesis showed that Glu_B_730 is a fundamental residue for the catalytic reaction by abstracting a proton from the Mo-OH group, which then nucleophilically attacks the substrate carbon atom to be hydroxylated [14]. Two other conserved residues at the active site of XORs, Glu_B_232 and Arg_B_310, are involved in substrate binding and transition state stabilization [14], [17]. In mouse AOX1 (mAOX1), the glutamate acting as an active site base is also highly conserved (E1265), however, the glutamate involved in substrate binding is exchanged by a valine (V806), and the arginine involved in transition state stabilization is exchanged to a methionine (M884) [3].

To study the importance of these amino acids in substrate specificity for AOX1, we established a system for heterologous expression of mAOX1 in *E. coli*. To ensure a high level of incorporation of the sulfido ligand at the Moco site, mouse MCSF (mMCSF) was cloned and coexpressed in this system. The recombinant enzyme was characterized by spectroscopic methods and steady state kinetics. Using site-directed mutagenesis, we exchanged the amino acids present at the active site of mAOX1 by the ones found in XOR. For comparison reasons, we introduced the reverse amino acid exchanges to the active site of *R. capsulatus* XDH. We determined the kinetic parameters of wild-type and mutant mAOX1 using different aldehyde and purine substrates, gaining insights into the molecular determinants guiding substrate specificity in XOR and AOX1.

## Results

### Heterologous Expression, Purification and Characterization of mAOX1

Several heterologous expression systems for mammalian AOs have been described. However a main drawback of all these systems is the low catalytic acivity of the purified enzymes due to a low Moco content [18]–[20]. To overcome this problem, we designed an expression system that allowed the simultaneous expression of mAOX1 and mMCSF in the *E. coli* TP1000 strain, and ensured a higher level of sulfurated Moco insertion into the enzyme [21].

**Figure 1:**
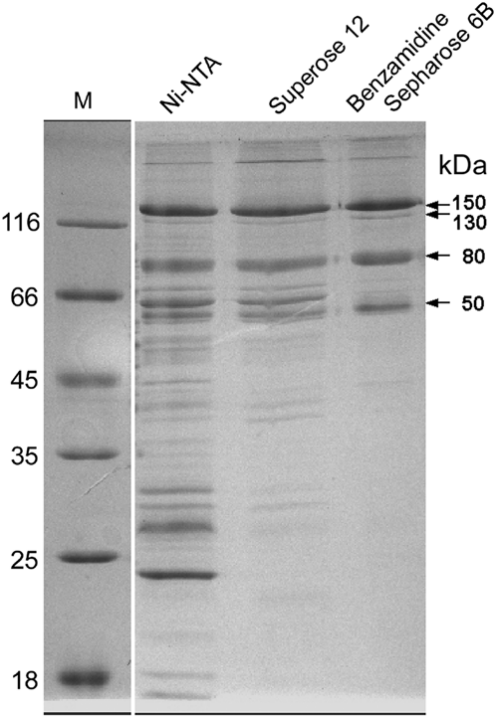
Purification of mAOX1 after heterologous expression in *E. coli*. 12% SDS-PAGE analysis of purification of mAOX1. The protein was purified after Ni-NTA, Superose 12 and benzamidine sepharose 6B. Purified mAOX1 displays a molecular mass of 150 kDa on SDS-PAGE, the three bands with sizes of 120 kDa, 80 kDa, and 50 kDa correspond to degradation products of mAOX1, as determined by mass spectrometry.

Recombinant mAOX1 was purified from a 12-liter *E. coli* culture using sequential Ni-NTA chromatography, size exclusion chromatography and benzamidine sepharose affinity chromatography. The purification protocol used for mAOX1 is summarized in Table 1. The overall purification from the soluble fraction was more than 23-fold with a yield of 0.17%, and the final specific activity with benzalyldehyde of the purified protein was 2.3 U/mg. This compares well to the activity reported for mAOX1 purified from mouse liver [7]. We demonstrated that the low increase in specific activity after Ni-NTA is due to the intrinsic aldehyde oxidizing activity of *E. coli* enzymes of the xanthine oxidase family present in the crude extract (data not shown). Size exclusion chromatography of the purified protein resulted in a single peak with an approximate molecular mass of 300 kDa, showing that the protein existed as a homodimer in solution (data not shown). SDS polyacrylamide gels performed under reducing conditions demonstrated the presence of one major band with a size of 150 kDa after purification (Fig. 1). In these experimental conditions three additional bands with sizes of 120 kDa, 80 kDa, and 50 kDa were also observed (Fig. 1). Electrospray mass spectrometry analysis of the respective gel slices revealed that these bands were degradation products of mAOX1 (data not shown). These bands were also observed in AO purified from other sources, e.g. in AO purified from rat liver [12]. As observed in our data, AO purified from rat liver displayed several bands of sizes of 150, 130, 80, and 45 kDa on SDS- polyacrylamide gels, but not after native-PAGE [12]. Thus we conclude, that the degradaion products occur due the reductive conditions during the SDS-PAGE.

**Table 1:** Purification of recombinant mAOX1 after expression in *E. coli* TP1000 cells.

Step	Volume	Protein^a^	Total^b^	Benzaldehyde oxidizing activity		
				S.A.^c^	Yield	P.F.
	ml	mg	units	units/mg	%	-fold
Cytosol	150	1611	135	0.1	100	1
Ni-NTA	14	6.6	0.76	0.12	0.57	1.2
Superose 12	15	0.84	0.35	0.43	0.27	4.2
BAS	20	0.1	0.23	2.3	0.17	23

a Total protein was quantified with the Bradford assay.

b The activity was measured by monitoring the decrease in absorption at 600 nm in the presence of 500 µM benzaldehyde and 100 µM DCPIP.

c Specific enzyme activity (units/mg) is defined as the oxidation of 1 µM benzaldehyde per min and mg of enzyme under the assay conditions.

**Figure 2:**
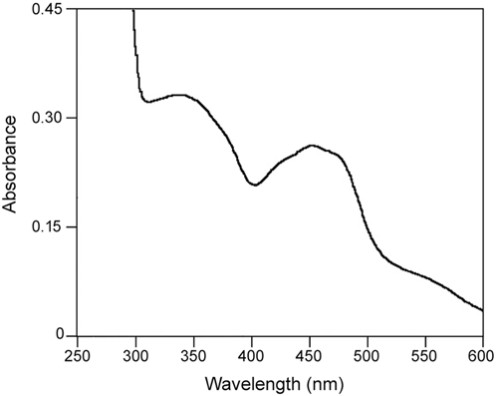
Characterization of wild-type mAOX1 by UV-VIS absorption spectroscopy. Spectra of 7 µM of the air-oxidized mAOX1 in 50 mM Tris, 1 mM EDTA, pH 7.5, under anaerobic conditions.

The visible absorption spectrum (Fig. 2) of recombinant mAOX1 is similar to those of mAOH1 purified from mouse liver and shows the presence of FeS and FAD as prosthetic groups [8]. The iron content was measured by ICP-OES and showed a saturation of 90% (Table 2). Since the ratio of 450∶550 in the UV-Vis spectrum was shown to be 3, this implicates that the protein is fully saturated with FAD [1]. The Moco content of mAOX1 was quantified after its conversion to Form A and related to the molybdenum content of the protein (Table 2), revealing that no demolybdo-mAOX1 was present in the purified fractions. To determine the content of the terminal sulfur ligand required for mAOX1 activity, the cyanolysable sulfur was quantified and in addition, absorption spectra of oxidized mAOX1 and benzaldehyde reduced enzyme were recorded under anaerobic conditions (data not shown). From the reduction spectra the amount of active mAOX1 was calculated to be 20%. While purified mAOX1 was 70% saturated with Moco (Table 2), the purified enzyme was only 20% saturated with the sulfido ligand required for enzyme activity.

**Table 2:** Determination of the Moco and iron content of mAOX1 and *R. capsulatus* XDH and variants.

Protein	Moco content^a^ %	Fe-content^b^ %
mAOX1:		
WT	70	90
V806E	46	101
M884R	66	97
V806E/M884R	44	108
E1265Q	60	83
Rc XDH:		
WT	75	99
E232V	43	95
R310M	63	88
E232V/R310M	49	97

a Moco was quanitfied as described in Materials and Methods. Moco content of wild-type mAOX1 and *R. capsulatus* XDH was set to the calculated molybdenum content determined by ICP-OES, and Moco determined as Form A in the AOX1 variants was compared to that value.

b Iron was determined by ICP-OES as described in Materials and Methods.

### EPR spectroscopy of the mAOX1 FeS clusters

**Figure 3:**
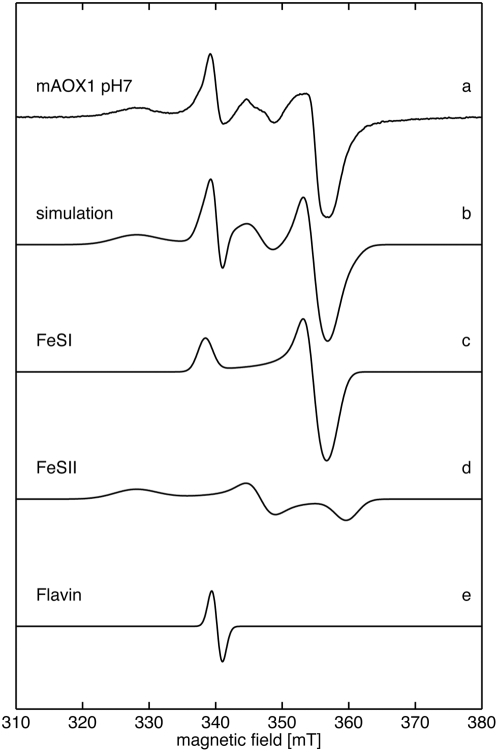
EPR spectra of mAOX1 wild-type. Experimental cw-EPR spectra of dithionite-reduced mAOX1 wild-type samples at pH 7.0 (trace a) together with the corresponding simulation (trace b). For simulation parameters see Table 3. The flavin semiquinone was simulated with an isotropic g-value of g_iso_ = 2.0 and 1.9 mT (trace e). (MoV) was neglected in all simulations. (a) mAOX1 wild-type; (b) simulation of complete spectrum; (c) simulation of FeSI; (d) simulation of FeSII; (e) simulation of FAD. Experimental conditions: T = 20 K, 1 mW microwave power, 1 mT modulation amplitude, 12.5 kHz modulation frequency.

Fig. 3 shows the EPR spectra of the FeS clusters of dithionite-reduced mAOX1 wild-type (trace a), together with the corresponding simulations (traces b–e). The spectra show signals from the reduced FAD cofactor to the flavin semiquinone and from some remaining Mo(V). Most prominent are however the characteristic EPR signals assigned to the two iron sulphur centers FeSI and FeSII, which are similar for all members of the xanthine oxidase family that have been described to date [1], [22]. FeSI has EPR properties showing an almost axial g-tensor, similar to those of many other [2Fe-2S] proteins, being fully developed at relatively high temperatures (60 K), while FeSII has unusual EPR properties for [2Fe-2S] species with a strongly rhombic g-tensor, showing broad lines and being only observed at much lower temperatures (20 K). The g-values and linewidths were evaluated by simulating the superimposed spectra of FeSI, FeSII and reduced FAD (Fig. 3) using the program *EasySpin* [23]. The double-integrated simulated spectra for the single iron-sulfur clusters display a ratio of 1∶1 indicating the presence of both clusters FeSI and FeSII in the same amount in the protein. The obtained g-values are given in Table 3. The flavin semiquinone (FAD) has been simulated by using an isotropic g-value of 2.0 and a linewidth of 1.9 mT. This linewidth is comparable with that from other flavins [1], [22]. The Moco (MoV) has been neglected in the simulations.

**Table 3:** EPR linewidths and g-values of FeSI and FeSII from mAOX1.

Protein	Cluster	g-values			linewidth [mT]
		g_x_	g_y_	g_z_	
Rabbit liver AO^a^	FeSI^a^	2.018	1.930	1.918	1.6
	FeSII^a^	2.106	2.003	1.915	-
mAOX1	FeSI^b^	2.019	1.927	1.912	2.6
	FeSII^c^	2.085	1.971	1.90	4.0

a AOX wild-type from rabbit liver, values from [36].

b g-strain was included in the simulation with 0.01 for g_z_ .

c g-strain was included in the simulation with 0.04 for g_x_ . Estimated error of g-values: ±0.004 for FeSI and ±0.008 for FeSII.

### CD-Spectroscopy

**Figure 4:**
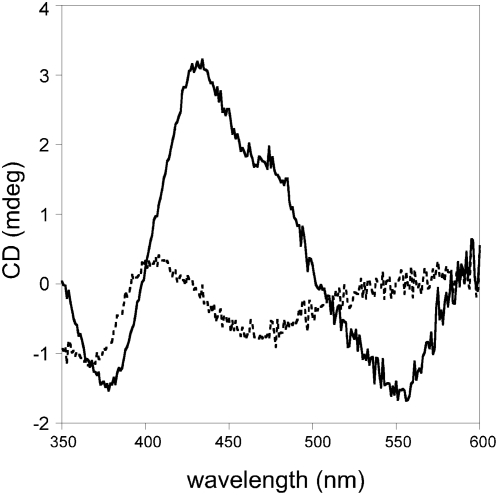
CD-Spectroscopy of mAOX1 wild-type. Spectra were recorded in 50 mM Tris, 1 mM EDTA, pH 7.5 at 10°C using a Jasco J-715 CD-spectrometer. Spectra of 2.1 mg/mL of mAOX1 were recorded in the oxidized state (solid lines) and after reduction with sodium dithionite (dotted lines).

CD-spectra were measured in the visible region in both the reduced and oxidized forms (Fig. 4). The spectrum of the oxidized wild-type enzyme exhibited strong negative dichroic bands at approximately 350–400 nm and 520–580 nm, and intensive positive bands between 400 and 500 nm (Fig. 4). From the various maxima and infections, transitions can be identified at 378 (−), 434 (+), 474 (+), and 556 (−) nm. Upon reduction with dithionite, the spectrum changed markedly with less intense transitions at 370 (−), 408 (+), and 478 (−) nm. The visible CD-spectra of reduced and oxidized mAOX1 are very similar in shape and intensity to those of XOR [24], [25], showing that both FeSI and FeSII are present in the recombinant enzyme.

### Steady state kinetics of mAOX1 wild-type and active site variants E1265Q, V806E, M884R, and V806E/M884R

**Figure 5:**
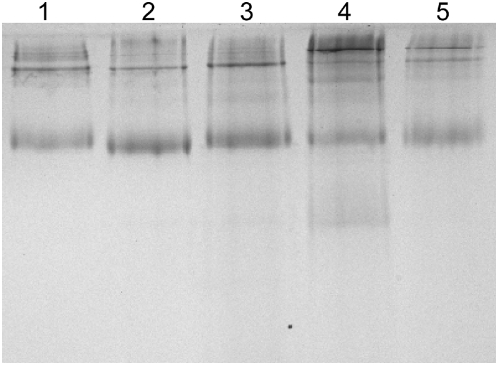
Native PAGE of mAOX1 wild-type and variants after purification. Purified enzymes were analyzed by 7% native PAGE. Each lane contained 6 µg of purified enzyme: lane 1, mAOX1 wild-type; lane 2, mAOX1-V806E; lane 3, mAOX1-M884R; lane 4, mAOX1-V806E/M884R; lane 5, mAOX1-E1265Q.

To determine the role of amino acids Glu1265, Val806, and Met884 for the oxidation of aldehydes by mAOX1, the variants E1265Q, V806E, M884R, and the double variant V806E/M884R were generated and purified. Fig. 5 shows wild-type mAOX1 with the purified variants on a native polyacrylamide gel, showing that all proteins had about the same purity.

Since some of the variants were inactive with benzaldehyde, the Moco content of the variants was determined in comparison to wild-type mAOX1, and showed the following range of Moco saturation: E1265Q 60%, V806E 46%, M884R 66%, and V806E/M884R 44% (Table 2). The lower Moco content of the purified variants may be due to the modified purification protocol necessary for the purification of these mAOX1 variants, which did not bind to the benzamidine sepharose. The iron content of the variants varied between 83–108%, reflecting that the FeS clusters were not influenced by the amino acid exchanges at the active site (Table 2). In addition, the variants showed almost identical CD-spectra in comparison to wild-type mAOX (data not shown).

With the exception of retinaldehyde, steady state kinetics of wild-type mAOX1 were performed by varying the concentrations of the substrates benzaldehyde, phtalazine, acetaldehyde, xanthine and hypoxanthine, and by using DCPIP as electron acceptor (Experimental procedures). In consideration of the fact that the protein was only 20% active, the enzyme activities compared well to the values obtained for native mAOX1 [7]. In particular the k_cat_ values determined with benzaldehyde were almost superimposable (Table 4). The k_cat_ values determined for retinaldehyde and acetaldehyde were a little higher in comparison to native mAOX1, but were in the same range. The substrates with the highest catalytic efficiency were the aromatic aldehydes phtalazine, benzaldehyde and retinaldehyde. Acetaldehyde showed the lowest catalytic efficiency. The purine substrates xanthine and hypoxanthine were not used by the recombinant mAOX1 under our assay conditions.

**Table 4:** Steady-state kinetic parameters of recombinant mAOX1 and variants with different aldehyde and purine substrates.

substrate	Kinetic parameters^a^	mAOX1-WT	V806E	M884R	V806E/M884R	E1265Q
benzaldehyde	*K_m_[µM]*	97.7±21.5	634.5±76.4	7130±2580	n.d.	n.d.
	*k_cat_[min^−1^]*	317.6±28.5	151.0±20.8	76.4±17.8	n.d.	n.d.
	*k_cat_/K_m_[µM^1^ min^−1^]*	3.34±0.56	0.24±0.02	0.011±0.002	-	-
phthalazine	*K_m_[µM]*	11.4±4.0	28.55±0.35	n.d.	n.d.	n.d.
	*k_cat_[min^−1^]*	128.1±13.0	103.0±1.0	n.d.	n.d.	n.d.
	*k_cat_/K_m_[µM^1^ min^−1^]*	12.2±4.0	3.6±0.1	-	-	-
retinaldehyde	*K_m_[µM]*	55.8±8.8	22.2±2.8	7.5±2.0	n.d.	n.d.
	*k_cat_[min^−1^]*	49.5±8.7	13.9±2.4	1.3±0.4	n.d.	n.d.
	*k_cat_/K_m_[µM^1^ min^−1^]*	0.89±0.02	0.63±0.04	0.17±0.02	-	-
acetaldehyde	*K_m_[µM]*	17500±4900	52900±17800	n.d.	n.d.	n.d.
	*k_cat_[min^−1^]*	519.9±112.3	160.6±38.1	n.d.	n.d.	n.d.
	*k_cat_/K_m_[µM^1^ min^−1^]*	0.030±0.002	0.0031±0.0003	-	-	-
xanthine	*K_m_[µM]*	n.d.	n.d.	n.d.	n.d.	n.d.
	*k_cat_[min^−1^]*	n.d.	n.d.	n.d.	n.d.	n.d.
	*k_cat_/K_m_[µM^1^ min^−1^]*	-	-	-	-	-
hypoxanthine	*K_m_[µM]*	n.d.	n.d.	n.d.	n.d.	n.d.
	*k_cat_[min^−1^]*	n.d.	n.d.	n.d.	n.d.	n.d.
	*k_cat_/K_m_[µM^1^ min^−1^]*	-	-	-	-	-

a Apparent kinetic parameters were recorded in 50 mM Tris, 1 mM EDTA, pH 7.5 by varying the concentration of substrate in the presence of 100 µM DCPIP as electron acceptor.

n.d., none was detectable.

-, not determined.

The catalytic parameters for the mAOX1 variants E1265Q, V806E, M884R, and V806E/M884R were also determined. As shown in Table 4, variants E1265Q and V806E/M884R were unable to metabolize any of the aldehyde and purine substrates tested. While the corresponding k_cat_ values were generally decreased, the K_m_ value for benzaldehyde, phtalazine and acetaldehyde were drastically increased by introducing the V806E amino acid exchange in mAOX1. For the variant V806E, the catalytic efficiency decreased by a factor of 10 for benzaldehyde, retinaldehyde and acetaldehyde, while the value was only decreased 3-fold for phtalzine as substrate. Thus, the affinity for small and symmetric aromatic aldehydes are affected by this mutation, while the affinity for more hydrophobic aldehydes like retinaldehyde was increased, albeit with a concomitant decrease in k_cat_. In contrast, the M884R variant was inactive with phtalazine and acetaldehyde as substrates, and for both benzaldehyde and retinaldehyde k_cat_ and K_m_ were decreased.

### Comparison of steady state parameters of the reverse amino acid exchanges (E_B_232V, R_B_310M, and E_B_232V/R_B_310M) introduced into *R. capsulatus* XDH

The catalytic mechanism of *R. capsulatus* XDH was proposed to involve three amino acids at the active site: Glu_B_730 is thought to act as an active site base in the initial step of the reaction, while Glu_B_232 is involved in substrate binding and transition state stabilization and R_B_310 is involved in transition state stabilization and orientation of the substrate at the active site. Since the catalytic mechanism of *R. capsulatus* XDH is well characterized, we compared the steady state kinetic data derived for wild-type mAOX1 and active-site variants with amino acid exchanges introduced into the active site of *R. capsulatus* XDH to reverse the amino acids found at the active site of XDH to the ones conserved in mAOX1. Thus, the XDH variants E_B_232V, R_B_310M, and the double variant E_B_232V/R_B_310M were generated.

As shown in Table 2, the Moco content of the purified XDH proteins was reduced and varied from 43%–63%, depending on the introduced amino acid exchange. Steady state kinetics of XDH were performed by varying the concentrations of the substrates benzaldehyde, phtalazine, retinaldehyde, acetaldehyde, xanthine and hypoxanthine, by using DCPIP as electron acceptor. Compared to wild-type XDH, the activity of the variants with purine substrates was drastically decreased, while oxidation of aldehydes as substrate was increased (Table 5). We were able to sucessfully purify the XDH-E_B_232V/R_B_310M double variant. This variant was devoid of purine oxidizing activity (Table 5), however, in contrast, aldehydes were oxidized with a higher turnover number in comparison to wild-type XDH. In general, the k_cat_ values of the three XDH variants for all aldehyde substrates tested was increased, while the K_m_ was decreased for benzadehyde and phtalazine and increased for retinaldehyde and acetaldehyde. Overall, the results obtained for *R. capsulatus* XDH show that the two amino acids at the active site switch the substrate specificity from purines to aldehydes. However, the reverse was not true for amino acids exchanges in the active site of mAOX1, showing that the oxidation of aldehydes is more complex.

**Table 5:** Steady-state kinetic parameters of recombinant *R. capsulatus* XDH and variants with different aldehyde and purine substrates.

substrate	Kinetic parameters^a^	XDH-WT	E232V	R310M	E232V/R310M
benzaldehyde	*K_m_[µM]*	540±146	39.9±7.0	552±188	25.9±15.5
	*k_cat_[min^−1^]*	33±2	93.5±13.6	211±53	94.2±8.4
	*k_cat_/K_m_[µM^1^ min^−1^]*	0.063±0.013	2.4±0.1	0.36±0.08	4.1±1.3
phthalazine	*K_m_[µM]*	1240±210	310±80	n.d.	420±73
	*k_cat_[min^−1^]*	2.62±0.06	142.0±27.4	n.d.	81.0±10.5
	*k_cat_/K_m_[µM^1^ min^−1^]*	0.0022±0.0004	0.47±0.04	-	0.20±0.02
retinaldehyde	*K_m_[µM]*	6.3±1.3	38.2±10.5	154.8±7.1	36.6±1.1
	*k_cat_[min^−1^]*	0.39±0.06	3.3±0.9	10.9±0.2	3.0±0.2
	*k_cat_/K_m_[µM^1^ min^−1^]*	0.063±0.005	0.087±0.003	0.071±0.003	0.082±0.005
acetaldehyde	*K_m_[µM]*	n.d.	913±460	6200±1880	2170±450
	*k_cat_[min^−1^]*	n.d.	88.5±20.1	110.6±10.9	56.8±7.6
	*k_cat_/K_m_[µM^1^ min^−1^]*	-	0.11±0.03	0.019±0.004	0.027±0.005
xanthine	*K_m_[µM]*	59±10	175±7	n.d.	n.d.
	*k_cat_[min^−1^]*	3300±460	85.6±15.3	n.d.	n.d.
	*k_cat_/K_m_[µM^1^ min^−1^]*	56.4±6.6	0.49±0.07	-	-
hypoxanthine	*K_m_[µM]*	31.4±1.4	n.d.	63.7±10.1	n.d.
	*k_cat_[min^−1^]*	2666±186	n.d.	9.0±0.3	n.d.
	*k_cat_/K_m_[µM^1^ min^−1^]*	84.8±3.4	-	0.14±0.03	-

a Apparent kinetic parameters were recorded in 50 mM Tris, 1 mM EDTA, pH 7.5 by varying the concentration of substrate in the presence of 100 µM DCPIP as electron acceptor.

n.d., none was detectable.

-, not determined.

## Discussion

Here, we report a system for the heterologous expression of mAOX1 in *E. coli*. Our system tried to overcome one of the main problems associated with the expression of recombinant mammalian MFEs in bacteria, i.e. the production of large proportions of inactive demolybdo and desulfo enzymes, as observed in the case of human XOR [20]. To ensure a higher sulfuration level of AOX1, we engineered *E. coli* for the simultaneous synthesis of the mMCSF and mAOX1 proteins. After coexpression with mMCSF, mAOX1 contained a 50% higher level of the terminal sulfido ligand of Moco (data not shown), although only 20% of the purified enzyme existed in the catalytically active form. This suggested two possibilities: either the conditions for the expression of both proteins have to be further optimized, or the sulfuration of Moco in bacteria and eukaryotes is different. Thus in prokaryotes, sulfuration of Moco precedes insertion into MFEs [26]. In eukaryotes, like *Arabidopsis thaliana* it was speculated that Moco sulfurase sulfurates Moco already bound to MFEs [27]. Thus it remains possible that due to this difference, heterologous expression of mammalian MFE's in *E. coli* will not give rise to a complete sulfurated enzyme. However, we also tried expression of AOX1 in *Pichia pastoris*, and this expression system also did not give rise to a higher sulfuration level of AOX1. Thus, it also remains possible, that part of the sulfido ligand of mAOX1 is exchanged to an oxo-ligand during purification of the enzyme.

The recombinant mAOX1 displayed similar catalytic properties in comparison to the enzyme purified from mouse liver [7]. This demonstrates that the protein was correctly folded in mMCSF engineered *E. coli* cells and could be used for more detailed analyses. The EPR spectra of mAOX1 were found to be very similar to those from the bacterial XDH characterized from *R. capsulatus*, showing a rather axial signal for FeSI [28]. There are only subtle differences in the g-values and linewidths, in particular for the FeSII center, that may be the consequence of small changes in bond distances and angles of the ligands of the respective FeS centers [28]. Nevertheless, the overall close similarity of the EPR parameters indicated the presence of the same ligands and similar geometries of the two redox centers in mAOX1 and *R. capsulatus* XDH. The spectroscopic charactaristics of mAOX1 and rabbit liver AO were also similar [29] and are consistent with an axial signal for FeSI.

Eukaryotic XOR and AO are similar in protein structure and prosthetic group composition, but have different characteristics of substrate specificity both at the molybdenum center and at the FAD center [3], [11]. While mAOX1 is a true oxidase using molecular oxygen as electron acceptor at the FAD site, XOR exists in two interconvertible forms: NAD^+^ is the substrate for the XDH form of XOR, while after proteolytic cleavage or intramolecular disulfide formation, the XO form is generated, which uses O_2_ instead of NAD^+^. The hydroxylation of aldehyde and purine substrates is catalyzed at the molybdenum center in both AO and XOR. However, the former catalyzes the hydroxylation of aldehydes more efficiently than the latter [11]. On the other hand, purines such as hypoxanthine and xanthine are good substrates of XOR, but poor substrates of AO. The crystal structure of *R. capsulatus* XDH with alloxanthine bound to the active site [15], in addtion to site directed mutagenesis studies [14], proposed that the purine substrates are bound and oxidized as follows: Catalysis is initiated by abstraction of a proton from the Mo-OH group by the conserved active site Glu_B_730, followed by the nucleophilic attack on the carbon of the substrate and the concomitant hydride transfer to the Mo = S of the molybdenum center [14]. This reaction yields an intermediate with the hydroxylated product coordinated to the molybdenum via the newly introduced hydroxyl group. The catalytic sequence is completed by displacement of the bound product from the molybdenum coordination sphere by hydroxide from solvent. This is followed by intramolecular electron transfer to the FeS and FAD cofactors, as well as deprotonation of the Mo-SH to give the oxidized form of the molybdenum center. In addition, Glu_B_232 and Arg_B_310 of *R. capsulatus* XDH were shown to be involved in the binding, orientation and transition state stabilization of the substrate [14], [17].

A similar reaction mechanism as the one described for XOR have been proposed for AOs [4], although, this proposal has never been supported by experimental data based on site-directed mutagenesis. Heterologous expression of mAOX1 in *E. coli* enabled us to study the involvement of specific residues at the active site in more detail for the first time. We exchanged residues Glu1265, Val806, and Met884 to the ones identified in the active site of XORs. For direct comparison reasons, the reverse amino acid exchanges were introduced to the active site of *R. capsulatus* XDH. Our results showed that the amino acid exchanges in XDH resulted in the complete loss of activity towards purine substrates for the E_B_232V/R_B_310M double variant, and a higher activity with aldehyde substrates. In contrast to Yamaguchi et al. [20], we were able to purify the E_B_232V/R_B_310M double XDH variant. However, the reverse was not the case for amino acid exchanges in mAOX1. The amino acid exchanges V806E/M884R in mAOX1 resulted in a complete loss of enzyme activity with both purine and aldehyde substrates. Both residues at the active site of mAOX1 seem to be important for the binding of substrates, since the M884R variant either completely lost the activity with phtalazine or acetaldehyde as substrates, or the activity with retinaldehyde and benzaldehyde was drastically reduced, with a concomitant increase of K_m_ for benzaldehyde. In general, the V806E exchange resulted in an increase in K_m_ for most substrates (except retinaldehyde) and in a decrease in k_cat_. Thus, while the ability to bind and utilize aldehyde substrates was decreased by converting the two residues to the ones found in XDH, purines were still not converted by the mAOX1 variants. While in XDH the two residues seem to determine which substrates are efficiently bound at the active site, in mAOX1 more factors determine the binding and conversion of substrates. mAOX1 seems to be more adapted to aldehyde substrates and the substrate specificity can not be converted back to purine substrates by two amino acid exchanges at the active site. The active site of XDH is deeply buried in the enzyme, but reachable through a funnel-shaped cavity that is wider on the surface. Hydrophobic residues, able to accommodate the ring structures of the aromatic substrates dominate the channel in XOR (e.g. Leu_B_303, Pro_B_306, Phe_B_344, Phe_B_459 in *R. capsulatus* XDH) [15]. These residues are exchanged to more bulky, charged or hydrophobic amino acids in mAOX1: Glu877, Trp880, Phe918, and Ile1013 [3]. It is possible that the aromatic purine residues analyzed in this study, are unable to enter the active site of mAOX1.

**Figure 6:**
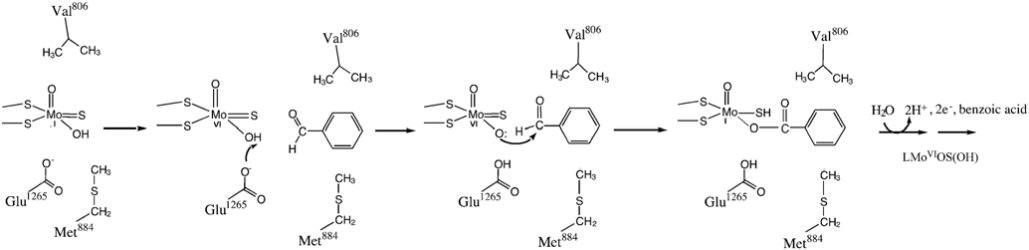
Proposed base catalyzed mechanism for mAOX1. E1265 acts as an active site base that abstracts a proton from the Mo-OH group, which in turn undertakes a nucleophilic attack on the substrate benzaldehyde. After hydride transfer to the Mo = S group, the initial intermediate breaks down, with the transient formation of a paramagnetic Mo^V^ species, followed by displacement of product by a water molecule to return to the starting LMo^VI^OS(OH) state. The roles of residues Met884 and Val806 are stabilization of substrate binding.

Finally, the mAOX1-E1265Q variant was catalytically inactive. This result is consistent with an essential catalytic role for this residue [4]. Like in XOR, Glu1265 of mAOX1 may function as an active-site base to abstract a proton from the Mo-OH leading to the nucleophilic attack on the aldehyde substrate molecule, which is followed by a hydride transfer to give the transition state intermediate (Fig. 6). The roles of residues Met884 and Val806 are proposed to be the stabilization of substate binding. Since the aromatic aldehydes tested in this study are symmetric, the orientation of the substrate at the active site is not as important as in XOR [17].

In total, the established expression system of mAOX1 in *E. coli* can further be used for detailed site-directed mutagenesis and structure-function studies of mammalian MFEs. In future studies it is planned to characterize the homologues of mAOX1 in rodents in more detail. Analysis of substrate specificities will give insights into the potential physiological roles of these enzymes.

## Materials and Methods

### Bacterial Strains, Plasmids, Media and Growth Conditions

*E.coli* TP1000 (Δ*mob*AB) cells [30] were used for the coexpression of mAOX1 wild-type and variants with mMCSF. *E. coli* expression cultures were grown in LB medium under aerobic conditions at 30°C for 24 h. Ampicillin (50 µg/ml) chloramphenicol (150 µg/ml), sodium molybdate (1 mM) and isopropyl-ß-D-thiogalactoside (IPTG) (20 µM) were used when required.

### Cloning Expression and Purification of mouse AOX1 and variants

The cDNA of mAOX1 was cloned from mouse CD1 liver [31], using primers designed to allow cloning into the *Nde*I-*Sal*I sites of the expression vector pTrcHis [32]. The resulting plasmid was designated pSL205, and expresses mAOX1 as a N-terminal fusion protein with a His_6_-tag. By using PCR mutagenesis the amino acid exchanges V806E, M884R, V806E/M884R and E1265Q were introduced into mAOX1. The mMCSF cDNA fragment was cloned from mouse liver using primers for the cloning into the *Nde*I-*Xho*I sites of the expression vector pACYCDuet-1 (Novagen), and the resulting plasmid was designated pSS110.

For heterologous expression in *E. coli*, pSL205 and pSS110 were transformed into TP1000 cells [30]. To express recombinant proteins, cells were grown at 30°C in LB medium supplemented with 150 µg/mL ampicillin, 50 µg/ml chloramphenicol, 1 mM molybdate, and 20 µM IPTG. After 24 h, cells were harvested by centrifugation, resuspended in 50 mM sodium phosphate buffer, pH 8.0, containing 300 mM NaCl and disrupted by several passages through a French Press cell. The supernatant was mixed with 3.5 mL of Ni-nitrilotriacetic (NTA) resin per 12 liters of cell growth. The slurry was transferred to a column and washed with 10 column volumes of 50 mM sodium phosphate, 300 mM NaCl, pH 8.0 containing 10 mM imidazole, followed by a wash with 10 column volumes of the same buffer containing 20 mM imidazole. His_6_-tagged mAOX1 was eluted with 50 mM sodium phosphate, 300 mM NaCl, pH 8.0 containing 250 mM imidazole. This fraction was dialyzed over night in 50 mM Tris-HCl, 200 mM NaCl, pH 7.5, containing 1 mM EDTA. The mAOX1 protein was further applied to a Superose 12 gel filtration column (GE Healthcare). The eluted fractions were analyzed by SDS-PAGE and the ones containing mAOX1 were combined and dialyzed against 100 mM Glycin, 200 mM NaCl, pH 9.0. The mAOX1 protein was mixed with 2 ml of benzamidine sepharose (GE Healthcare), pre-equilibrated in the same buffer. Following 4 h of incubation, the resin was washed 3 times with 10 ml of the equilibration buffer to remove unbound proteins. Absorbed proteins were eluted with 20 ml of equilibration buffer containing 30 mM benzamidine. Subsequently, the eluted protein was dialyzed into 50 mM Tris-HCl, pH 7.5 containing 1 mM EDTA. The protein solution was then concentrated by ultrafiltration. Absorbance spectra were recorded with a Shimadzu UV-2401 dual-wavelength double-beam spectrophotometer. Purified proteins were stored at −70°C until use. The mAOX1 variants were expressed under the same conditions as the mAOX1 wild-type and purified by Ni-NTA and Superose 12 chromatography.

### Site-directed mutagenesis and purification of R. capsulatus XDH and variants

*R. capsulatus* wild-type XDH was purified as described previously [14]. Using PCR mutagenesis amino acid exchanges E_B_232V, R_B_310M, and the double variant E_B_232V/R_B_310M were introduced into *R. capsulatus* XDH. The generated XDH variants were purified by Ni-NTA, Q-Sepharose, and Superose 12 chromatography as described previously for other XDH variants [14]. The purified enzymes were concentrated by ultrafiltration, gel filtered using a PD-10 gel filtration column (GE Healthcare) equilibrated with 50 mM Tris, 1 mM EDTA, 2.5 mM DTT, pH 7.5 and stored at −70°C until used.

### SDS-polyacrylamide Gel Electrophoresis (PAGE)

SDS-PAGE was performed as described by Laemmli [33] using 12% polyacrylamide gels. The gels were stained with Coomassie Brilliant Blue R.

### Enzyme assays

Enzyme assays were carried out at 30°C in Tris buffer (50 mM, 1 mM EDTA, pH 7.5) in a final volume of 500 µL. Total enzyme concentration was 30 nM for wild-type mAOX1 and 30–200 nM for the variants. Enzyme activity was monitored spectrophotometrically at 600 nm with 100 µM 2,6-dichlorphenolindophenol (DCPIP) as electron acceptor and calculated using the extinction coefficient of 16.1 mM^−1^ cm^−1^ for DCPIP [34].

To determine retinaldehyde oxidase activity, the assay described by Vila *et al.* [7] was used with some modifications. Purified mAOX1 (2 µg) was incubated in the dark for 10 min at 30°C in 100 µL of 10 mM potassium phosphate buffer, pH 7.4 and the reaction was started by the addition of 0.01–10 mM all-*trans*-retinaldehyde. The reaction was stopped with 100 µL of butanol/methanol (95∶5 v/v) containing 0.005% w/v of butylated hydroxytoluene (0.005% w/v) (Sigma-Aldrich). The organic phase was separated and 20 µL were subjected to high performance liquid chromatography on a C18 reverse phase column (4.6×250-mm ODS Hypersil, 5 µm). The production of the oxidation product all-*trans*-retinoic acid was determined at 340 nm and quantified by using a calibration curves.

### Absorption Spectra during Anaerobic Reduction

Purified mAOX1 in 150 µL of 50 mM Tris, 1 mM EDTA, pH 7.5, was incubated in an anaerobic chamber (Coy Lab Systems) for 2 h at 4°C before benzaldehyde was added to a final concentration of 300 µM. Complete reduction was achieved by the addition of 20 mM sodium dithionite. Spectra were recorded in 0.15 mL cuvettes using a Shimadzu UV-2401 PC spectrophotometer.

### Metal and Moco/MPT analysis

The molybdenum and iron contents of the purified proteins were quantified by ICP-OES analysis with a Perkin-Elmer Optima 2100 DV. The samples were wet-ashed at a concentration of 4 µM in a volume of 500 µL by the addition of 500 µM 65% nitric acid and incubated over night at 100°C. The samples were further diluted by the addition of 4 mL of water. As reference, the multi-element standard solution XVI (Merck) was used. The complete Moco content of mAOX1 in comparison to the variants was quantified after conversion to its fluorescent degradation product Form A as described earlier [35].

### CD-spectroscopy

UV/visible CD spectra of 2.1 mg/mL enzyme samples were recorded in 50 mM Tris, 1 mM EDTA, pH 7.5 using a Jasco J-715 CD-spectrophotometer. The scanning mode was set step-wise, each nm a data pitch was recorded, the response time was 2 seconds and each measurement was repeated 4 times.

### Electron Paramagnetic Resonance Spectroscopy

9.5 GHz X-band EPR spectra were recorded on a Bruker ESP300E spectrometer equipped with a TE_102_ microwave cavity. For temperature control between 5 K und 300 K an Oxford ESR 900 helium flow cryostat with an Oxford ITC4 temperature controller was used. The microwave frequency was detected with an EIP frequency counter (Microwave Inc.). Magnetic field was calibrated using a LiLiF standard with a known g-value of 2.002293±0.000002 [36]. Samples (typically 0.1 mM enzyme) were prepared in quartz tubes with 4 mm outer diameter. Chemical reduction, in order to generate the reduced Fe(II)/Fe(III) in the FeS clusters, was performed by adding a small volume of anaerobic sodium dithionite solution to the protein solution under a weak stream of argon gas (20-fold excess dithionite with respect to the protein). The sample tubes were frozen, after a change of colour was observed (typically 30 s reaction time) in liquid nitrogen. Baseline corrections, when required, were performed by subtracting a background spectrum, obtained under the same experimental conditions from a sample containing only a buffer solution. Simulations of the experimental EPR spectra, based on a spin Hamilton operator approach, were performed with the program *EasySpin* [23]. Second integrals from the simulated spectra of reduced FeSI and FeSII were used to estimate the relative amount of both clusters in the respective samples.
